# Investigation of red blood cell and platelet indices in adult dogs suffered from myxomatous mitral valve disease with and without pulmonary hypertension

**DOI:** 10.3389/fvets.2023.1234768

**Published:** 2023-09-15

**Authors:** Nattawan Tangmahakul, E. Christopher Orton, Sirilak Disatian Surachetpong

**Affiliations:** ^1^Department of Veterinary Medicine, Faculty of Veterinary Science, Chulalongkorn University, Bangkok, Thailand; ^2^Department of Clinical Sciences, College of Veterinary Medicine and Biomedical Science, Colorado State University, Fort Collins, CO, United States

**Keywords:** cardiology, myxomatous mitral valve disease, platelet distribution width, pulmonary hypertension, red cell distribution width

## Abstract

**Background:**

Pulmonary hypertension (PH) is a common complication of cardiopulmonary disease. In dogs, PH commonly occurs secondary to myxomatous mitral valve disease (MMVD). Red blood cell and platelet indices including mean corpuscular volume (MCV), mean corpuscular hemoglobin (MCH), mean corpuscular hemoglobin concentration (MCHC), red cell distribution width (RDW), mean platelet volume (MPV) and platelet distribution width (PDW), have previously been found to be indicators for predicting and prognosing PH in humans. Therefore, this study aimed to investigate whether these indices are associated with MMVD and/or PH in dogs.

**Methods:**

Two hundred and forty-six dogs were retrospectively recruited for the study and classified into 4 groups: normal (*n* = 49), MMVD (*n* =102), PH (*n* =17), MMVD+PH (*n* =78). A sub-analysis was performed in dogs with MMVD without evidence of PH according to stage B1 (*n* =20), stage B2 (*n* =15), stage C (*n* =67). The data are expressed as median (interquartile range).

**Results and discussion:**

No significant differences (*p* < 0.05) were found in MCV, RDW and MPV among all groups (normal, MMVD, PH and MMVD+PH). However, decreases in MCH and MCHC were found in MMVD [22.40 (20.90-23.50) pg and 35.25 (33.08-36.90) g/dL], MMVD+PH [22.25 (20.85-23.98) pg and 35.65 (33.30-37.33) g/dL] and PH groups [21.20 (20.60-22.20) pg and 33.80 (32.75-35.70) g/dL] compared to the normal dogs [24.29 (23.55-24.90) pg and 38.20 (37.50-39.05) g/dL] (*p* < 0.001). Decreases in PDW were found in dogs in the MMVD+PH [15.10 (14.98-15.30) %] groups compared to dogs in the normal group [15.30 (15.10-15.50) %] (*p* = 0.004). Sub-analysis of MMVD dogs without PH showed a decrease in MCH in dogs with stage B2 MMVD [21.00 (20.50-22.90) pg] and stage C MMVD [22.40 (20.90-23.20) pg] compared to normal dogs [24.29 (23.55-24.90) pg] (*p* < 0.001). MCHC of dogs with stage B1 [36.55 (33.53-37.78) g/dL] (*p* = 0.004), B2 [32.90 (32.00-35.00) g/dL] (*p* < 0.001) and C MMVD [35.30 (33.30-36.80) g/dL] (*p* < 0.001) were lower than those of normal dogs [38.20 (37.50-39.05) g/dL]. PDW in the stage C MMVD group [15.10 (15.00-15.30) %] was reduced compared to the normal group [15.30 (15.10-15.50) %] (*p*  = 0.042) and the stage B1 MMVD group [15.35 (15.23-15.68) %] (*p* = 0.002). MCH, MCHC and PDW were negatively correlated with the left atrial and left ventricular size.

**Conclusion:**

Decreases in MCH and MCHC are related to MMVD, precapillary PH and postcapillary PH while PDW are associated with MMVD severity but not with the presence of PH.

## Introduction

The consensus statement guideline of the American College of Veterinary Internal Medicine categorizes pulmonary hypertension (PH) into six groups: pulmonary arterial hypertension, PH due to left heart disease, PH secondary to respiratory disease/hypoxia, pulmonary emboli/thrombi/thromboembolism, parasitic disease, and PH with multifactorial or unclear mechanism (1). In older small breed dogs, PH commonly occurs secondary to myxomatous mitral valve disease (MMVD) (2). However, PH is also commonly found as a complication of respiratory and heartworm disease in dogs (3–5). Several studies in human patients have focused on the relationship between red blood cells (RBC) and platelet indices and disease progression and prognosis (6–14).

Red cell distribution width (RDW) measures size-heterogeneity of the circulating red blood cells (15). Red cell distribution width can be routinely reported as part of the complete blood count. This parameter is altered in various diseases such as anemia (16), cancer (17), and inflammatory diseases (18). Red cell distribution width has also been mentioned as a predictive and prognostic biomarker for heart failure, myocardial infarction, and PH in human patients because it positively correlates with the adverse outcomes of these cardiovascular diseases (6, 7, 9, 19, 20). The studies in human patients with heart failure showed that RDW had a positive correlation with N-terminal pro B-type natriuretic peptide, a powerful prognostic biomarker for heart failure (6). Moreover, increased RDW was related to the progression of complications and a decrease in survival time (7, 19). The study in human patients with PH revealed that an elevated RDW decreased the survival time (9). Consequently, RDW was a predictive or prognostic marker for morbidity and mortality of heart failure. An association between elevated RDW and adverse outcome has also been noted in dogs with MMVD (21). Therefore, RDW may be a candidate for detecting the progression of cardiovascular disease. RBC indices can be used to differentiate the type of anemia (22). Mean corpuscular volume (MCV) quantifies the average volume of RBC, while mean corpuscular hemoglobin (MCH) indicates the calculated hemoglobin amount per a single RBC, and mean corpuscular hemoglobin concentration (MCHC) reflects the calculated hemoglobin concentration in RBC (22, 23). Changes in RBC count, hemoglobin, hematocrit, MCH, MCHC, and RDW were reported previously in human with PH (24). Furthermore, negative correlation between RDW and MCV was found in dogs with MMVD (21).

Platelets play an important role in the inflammatory process because activated platelets secrete various inflammatory mediators (25, 26). The determination of platelet indices such as mean platelet volume (MPV) and platelet distribution width (PDW) can be routinely performed using an automated hematologic analyzer (25). The MPV reflects platelet production, activation, and function. PDW provides information on the variability of platelet size, which may also reflect platelet activation. Increased MPV and PDW have been found in several diseases associated with inflammatory conditions, including many cardiac diseases (27). Increases in MPV and PDW have been associated with increased severity of heart disease, heart failure, and adverse progressive cardiac outcome in human patients (10, 12, 28, 29). MPV was increased in human patients with mitral regurgitation (MR) from various causes and correlated with the severity of MR (13). An increased PDW was found in patients with heart failure (12). Furthermore, increased MPV and PDW were observed in patients with left-sided heart disease (28). MPV and PDW were prognostic biomarkers for cardiac diseases and heart failure. In addition, increases in these platelet indices have been noted in patients with PH (8, 11, 14). An increased MPV was revealed in various causes of PH (8). Elevated MPV and PDW were found in human patients with precapillary PH, and MPV may be a prognostic marker for PH due to positive association with the severity of PH (11, 14).

A study of RDW in dogs with PH has been published (30, 31). However, there is no study investigating both red blood cell and platelet indices that primarily focuses in MMVD dogs with PH. Therefore, this study aimed to investigate the changes in MCV, MCH, MCHC, RDW, MPV and PDW in normal dogs and dogs with PH or MMVD, or both.

## Materials and methods

### Animals

Data of dogs were retrieved retrospectively from the electronic medical records of the Small Animal Teaching Hospital, Faculty of Veterinary Science, Chulalongkorn University, Thailand. Signalment, history, physical, radiographic and echocardiographic findings, and hematologic and blood chemical profiles were noted. Since MMVD commonly occurs in senior small breed dogs, the inclusion criteria included dogs older than 6 years and weighing up to 15 kg. To assess the changes in RBC and platelet indices in dogs with PH, the dogs were divided into four groups of healthy dogs (normal group), dogs with MMVD (MMVD group), dogs with MMVD and PH (MMVD+PH group), and dogs with PH caused by other causes (PH group). The normal group consisted of healthy dogs that attended the Small Animal Teaching Hospital for a health checkup, and no abnormalities and diseases were found on physical examination, radiography, and echocardiography. Based on the criteria for PH secondary to left-sided heart disease (1), only dogs with left heart enlargement or MMVD stage B2 or greater were included in the MMVD+PH group. To clarify whether the severity of MMVD affected the change in RBC and platelet indices, a sub-analysis was performed in MMVD dogs without evidence of PH according to stage B1, stage B2 and stage C.

### Diagnosis and staging of MMVD and PH

The diagnosis and staging MMVD and PH were performed following the American College of Veterinary Internal Medicine guidelines (1, 32). Briefly, dogs were diagnosed with MMVD based on clinical presentation, radiography, and echocardiography. Dogs with stage B1 MMVD had mitral regurgitation without evidence of congestive heart failure (CHF) and structural changes in the heart. Dogs with stage B2 MMVD had left atrium to aorta ratio (LA/Ao) in the right parasternal short-axis view in early diastole >1.6, normalized left ventricular internal dimension at end-diastole (LVIDd) >1.7, and mitral regurgitation without evidence of heart failure. Dogs with stage C MMVD had mitral regurgitation, left atrial and ventricular enlargement, and previous history of pulmonary edema. Dogs with stage C MMVD responded to standard treatment with furosemide, spironolactone, angiotensin- converting enzyme inhibitor and pimobendan. Dogs with stage D MMVD exhibited clinical signs that were unresponsive to standard treatment of stage C MMVD and required additional medications such as antiarrhythmic drug and other diuretics (32). The underlying causes of PH were evaluated and classified. The peak tricuspid regurgitation (TR) velocity and the number of different anatomic sites with echocardiographic signs of PH were used to identify PH which included from low (≤3.0 m/s peak TR velocity, and no or 1 different anatomic site with echocardiographic signs of PH) to high (≤3.0 m/s peak TR velocity with 3 different anatomic sites of echocardiographic signs, 3.0–3.4 m/s peak TR velocity with ≥2 different anatomic sites of echocardiographic signs, or > 3.4 m/s peak TR velocity with ≥1 different anatomic site of echocardiographic signs of PH) probability of PH (1). The anatomic sites of echocardiographic signs of PH were investigated in the ventricles, pulmonary artery, and right atrium and caudal vena cava (1). Dogs with pregnancy and/or other systemic and inflammatory diseases, diagnosed through history taking, physical examination, hematology, and radiography were excluded.

### Red blood cell indices, platelet indices, and echocardiographic data collection

Hematological and blood chemistry profiles, and echocardiographic data were collected. The complete blood count and blood chemistry were performed by automated analyzers (BC-5000 Vet and BS-800, Mindray, China). The recorded complete blood count included red blood cell (RBC) count, hematocrit, MCV, MCH, MCHC, RDW, platelet count, MPV, PDW, and white blood cell (WBC) count. Thin blood smears were performed for screening blood morphology. Echocardiographic examination was conducted by an investigator (SS) utilizing a 4–12 MHz phased array transducer and an ultrasound machine (M9, Mindray, China). Echocardiographic data included left atrial size (LA) size, aorta size (Ao), the ratio of the left atrial dimension to the aortic annulus dimension, interventricular septum thickness at end-diastole (IVSd), LVIDd, left ventricular posterior wall thickness at end-diastole (LVPWd), interventricular septum thickness at end-systole (IVSs), left ventricular internal dimension at end-systole (LVIDs), left ventricular posterior wall thickness at end-systole (LVPWs), fractional shortening (FS), peak TR velocity, and calculated pulmonary arterial pressure (PAP).

### Statistical analysis

The computer-based program, SPSS version 22 (IBM, United States) was used for statistical analysis. Data distribution was analyzed with the Shapiro–Wilk test. Differences in hematologic and echocardiographic data between groups were assessed with the Kruskal-Wallis and Dunn post-hoc tests. The Mann–Whitney U test was used to analyze the differences in peak TR velocity between the MMVD+PH and PH groups. The receiver operating characteristic (ROC) curves with area under curve (AUC) were performed to determine whether the significant RBC and platelet indices could predictor MMVD and PH. An AUC value indicates the discriminatory power of these indices, where an AUC ≤ 0.75 implies no clinical utility, 0.75 < AUC < 0.97 indicates a moderately discriminative value, and an AUC of 0.97 represents an extremely clinical value (33). The optimal cut-point values for these significant RBC and platelet indices were calculated using Youden’s index and were used to estimate the sensitivity and specificity (34). Spearman’s rank correlation and multivariable regression analysis were used to examine the relationship between the quantitative and qualitative variables, respectively. Evidence of a difference for all tests was at *p* < 0.05. All data were expressed in median (interquartile range).

## Results

### Animals

The normal group (*n* = 49) consisted of 21 males and 28 females, including 21 Shih Tzus, 12 Chihuahuas, 6 Yorkshire Terriers, 3 Pomeranians, 1 Dachshund, and 6 mixed breeds. The MMVD group (*n* = 102) was composed of 20 dogs with stage B1, 15 dogs with stage B2 and 67 dogs with stage C MMVD. This group comprised 62 males and 40 females, with 31 Chihuahuas, 19 Pomeranians, 15 Poodles, 8 Shih Tzus, 4 Yorkshire Terriers, 2 Maltese, 2 Miniature Pinschers, 1 Beagle, 1 Chinese Crested Hairless Dog, 1 Finnish Splitz, 1 Miniature Schnauzer, 1 Dachshund and 16 mixed breeds. The MMVD+PH group (*n* = 78) included 7 dogs with stage B2, 68 dogs with stage C, and 3 dogs with stage D MMVD and PH. This group had 7 dogs with low probability of PH, 51 dogs with intermediate probability of PH, and 20 dogs with high probability of PH. There were 41 males and 37 females in the MMVD+PH group including 26 Chihuahuas, 14 Pomeranians, 10 Poodles, 9 Shih Tzus, 2 Miniature Pinchers, 1 Jack Russell Terrier, 1 Maltese, 1 Shetland Sheepdog, and 14 mixed breeds. The PH group (*n* = 17) included 14 dogs with PH due to respiratory problems and 3 dogs with PH due to heartworm disease. This group had 2 dogs with low probability of PH, 13 dogs with intermediate probability of PH, and 2 dogs with high probability of PH. There were 10 males and 7 females with 7 Pomeranians, 5 Chihuahuas, 1 Miniature Pincher, 1 Shih Tzu, 1 Jack Russell Terrier, 1 French Bulldog and 1 mixed breed dog. The age of the normal dogs was lower than that of the other groups (*p* < 0.05). No significant difference in age was found between the disease groups (Tables 1, 2).

**Table 1:** Signalment, hematological profiles, and echocardiographic data of dogs in the present study.

	Normal (*n* = 49)	MMVD (*n* = 102)	MMVD + PH (*n* = 78)	PH (*n* = 17)	*p-*value
Age (years)	8.00 (7.00–10.00)	12.00 (10.00–13.00)^a^	11.50 (10.00–13.00)^a^	11.00 (9.00–13.50)^a^	<0.001
Gender (M/F)	21/28	62/40	41/37	10/7	
Weight (kg)	5.50 (2.92–6.80)	4.32 (3.40–5.50)	4.62 (3.34–6.06)	4.00 (2.50–5.95)	0.215
RBC (×10^6^ cells/μL)	6.57 (6.16–7.44)	7.15 (6.39–7.62)	6.77 (6.01–7.54)	7.12 (6.11–7.94)	0.120
Hematocrit (%)	42.80 (38.80–47.00)	44.85 (40.00–48.50)	42.60 (38.55–46.50)	43.20 (39.10–48.45)	0.124
MCV (fL)	63.80 (61.80–64.95)	63.95 (60.90–65.78)	63.80 (60.58–66.58)	62.80 (60.30–66.25)	0.901
MCH (pg)	24.29 (23.55–24.90)	22.40 (20.90–23.50)^a^	22.25 (20.85–23.98)^a^	21.20 (20.60–22.20)^a^	<0.001
MCHC (g/dL)	38.20 (37.50–39.05)	35.25 (33.08–36.90)^a^	35.65 (33.30–37.33)^a^	33.80 (32.75–35.70)^a^	<0.001
RDW (%)	14.00 (13.05–14.55)	13.85 (13.10–14.70)	14.10 (13.20–15.03)	14.10 (13.45–14.45)	0.480
Platelet count (×10^3^ cells/μL)	283.00 (228.50–341.00)	325.50 (257.25–414.00)	395.00 (294.50–468.75)^a^	389.00 (283.00–454.00)	0.001
MPV (fL)	10.10 (9.05–11.35)	10.70 (9.70–11.40)	10.35 (9.70–11.33)	11.00 (9.15–12.50)	0.233
PDW (%)	15.30 (15.10–15.50)	15.20 (15.00–15.40)	15.10 (14.98–15.30)^a^	15.20 (15.00–15.40)	0.008
WBC (×10^3^ cells/μL)	8.37 (6.98–10.82)	8.63 (7.42–10.96)	9.59 (7.79–11.65)	8.79 (7.15–10.80)	0.230
Neutrophil (×10^3^ cells/μL)	5.87 (4.05–7.31)	6.09 (4.95–7.69)	6.98 (5.29–8.58)	6.51 (5.56–8.16)	0.064
Eosinophil (×10^3^ cells/μL)	0.31 (0.20–0.30)	0.31 (0.21–0.48)	0.37 (0.22–0.54)	0.28 (0.22–0.43)	0.848
Basophil (×10^3^ cells/μL)	0.02 (0.01–0.03)	0.01 (0.00–0.02)^a^	0.01 (0.00–0.02)^a^	0.01 (0.00–0.02)	0.006
Lymphocyte (×10^3^ cells/μL)	1.53 (1.18–1.94)	1.33 (1.06–1.77)	1.44 (1.11–1.87)	1.32 (1.00–1.79)	0.393
Monocyte (×10^3^ cells/μL)	0.57 (0.44–0.83)	0.50 (0.25–0.76)	0.49 (0.21–0.89)	0.44 (0.17–0.88)	0.071
LA (cm/kg)	0.92 (0.81–1.04)	1.96 (1.62–2.42)^a,c^	1.90 (1.53–2.43)^a,c^	1.43 (1.33–1.75)^a^	<0.001
Ao (cm/kg)	0.76 (0.68–0.86)	1.15 (0.91–1.32)^a^	0.97 (0.79–1.15)^a,b^	1.04 (0.96–1.18)^a^	<0.001
LA/Ao	1.20 (1.11–1.35)	1.75 (1.47–2.15)^a,c^	2.10 (1.77–2.40)^a,b,c^	1.43 (1.22–1.67)	<0.001
IVSd (cm/kg)	0.47 (0.40–0.52)	0.43 (0.38–0.49)	0.42 (0.36–0.49)	0.47 (0.41–0.59)	0.053
LVIDd (cm/kg)	1.22 (1.17–1.37)	1.77 (1.50–1.94)^a,c^	1.83 (1.65–2.00)^a,c^	1.30 (1.19–1.51)	<0.001
LVPWd (cm/kg)	0.38 (0.33–0.43)	0.40 (0.36–0.46)	0.41 (0.35–0.46)	0.44 (0.36–0.49)	0.181
IVSs (cm/kg)	0.61 (0.52–0.67)	0.64 (0.56–0.72)	0.68 (0.59–0.77)^a^	0.60 (0.56–0.74)	0.011
LVIDs (cm/kg)	0.74 (0.62–0.80)	0.88 (0.74–1.01)^a,c^	0.86 (0.69–1.04)^a^	0.76 (0.61–0.85)	<0.001
LVPWs (cm/kg)	0.64 (0.56–0.71)	0.67 (0.60–0.75)	0.69 (0.59–0.79)	0.59 (0.53–0.69)	0.018*
%FS	39.86 (34.75–45.68)	47.49 (41.86–53.12)	51.13 (44.50–55.47)^a^	47.50 (38.67–50.47)^a^	<0.001
TR (m/s)	–	–	3.70 (3.29–4.32)^c^	3.52 (2.86–3.76)	0.017
Calculated PAP (mmHg)	–	–	54.97 (43.24–74.62)^c^	49.51 (32.82–56.55)	0.017

Values are median (interquartile).

Ao, aorta; FS, fractional shortening; IVSd, interventricular septum thickness at end-diastole; IVSs, interventricular septum thickness at end-systole; LA, left atrium; LA/Ao, the ratio of the left atrial dimension to the aortic annulus dimension; LVIDd, left ventricular internal dimension at end-diastole; LVIDs, left ventricular internal dimension at end-systole; LVPWd, left ventricular posterior wall thickness at end-diastole; LVPWs, left ventricular posterior wall thickness at end-systole; MCH, mean corpuscular hemoglobin; MCHC, mean corpuscular hemoglobin concentration; MCV, mean corpuscular volume; MPV, mean platelet volume; PAP, pulmonary arterial pressure; PDW, platelet distribution width; RBC, red blood cell; RDW, red cell distribution width; TR, tricuspid regurgitation; WBC, white blood cell.

The p values represent the significant difference among 4 groups by the Kruskal-Wallis test.

The peak TR velocity was analyzed by Mann–Whitney U test.

*No significant difference was found after analysis with Dunn’s test.

^a^Indicates significant difference from the normal group.

^b^Indicates significant difference from the MMVD group.

^c^Indicates significant difference from the PH group.

**Table 2:** Blood chemistry profiles of dogs in the present study.

	Normal (*n* = 49)	MMVD (*n* = 102)	MMVD + PH (*n* = 78)	PH (*n* = 17)	*P-*value
ALT (U/L)	45.00 (34.00–56.50)	54.00 (37.00–80.75)	56.50 (36.00–90.00)	53.00 (38.00–69.00)	0.050
ALP (U/L)	50.00 (24.00–87.00)	56.50 (38.00–100.00)	67.00 (45.00–119.75)	69.00 (37.50–143.00)	0.047*
BUN (mg/dL)	17.60 (13.95–23.25)	25.30 (19.18–32.90) ^a^	31.00 (22.93–39.68)^a,c^	19.50 (10.60–31.00)	<0.001
Creatinine (mg/dL)	0.80 (0.70–0.90)	0.80 (0.60–1.00)	0.85 (0.70–1.10)^c^	0.60 (0.50–0.90)	0.017
Total protein (g/dL)	6.40 (6.00–6.90)	6.70 (6.40–7.10)	6.70 (6.03–7.00)	6.90 (6.25–7.30)	0.061
Albumin (g/dL)	3.20 (3.00–3.50)	2.80 (2.60–3.20)^a,c^	2.80 (2.50–3.00)^a^	2.60 (2.42–2.67) ^a^	<0.001

Values are median (interquartile).

ALP, alkaline phosphatase; ALT, alanine aminotransferase; BUN, blood urea nitrogen.

*No significant difference was found after analysis with Dunn’s test.

^a^Indicates significant difference from the normal group.

^b^Indicates significant difference from the MMVD group.

^c^Indicates significant difference from the PH group.

Echocardiographic results showed the greater LA/Ao and LVIDd in the MMVD and MMVD+PH groups compared with the normal and PH groups (*p* < 0.001). The peak TR velocity and calculated PAP of MMVD+PH was greater than those of PH group (*p* = 0.017) (Table 1). In sub-analysis of MMVD group, the greater LA/Ao, LVIDd and %FS were found in dogs with stage B2 and C MMVD compared with the normal dogs (*p* < 0.001). Moreover, LA/Ao and LVIDd, and %FS of dogs with stage C MMVD were greater than those of dogs with stage B1 MMVD (*p* < 0.001) (Table 3).

**Table 3:** Signalment, hematological profiles and echocardiographic data of dogs with myxomatous mitral valve disease.

	Normal (*n* = 49)	MMVD B1 (*n* = 20)	MMVD B2 (*n* = 15)	MMVD C (*n* = 67)	*P*-value
Age (years)	8.00 (7.00–10.00)	11.00 (8.25–13.00) ^a^	11.00 (10.00–13.00)^a^	12.00 (10.00–13.00)^a^	<0.001
Gender (M/F)	21/28	9/11	13/2	40/27	
Weight (kg)	5.50 (2.92–6.80)	4.09 (2.50–5.95)	3.80 (3.25–4.90)	4.60 (3.50–5.80)	0.240
RBC (×10^6^ cells/μL)	6.57 (6.16–7.44)	6.95 (6.35–7.55)	7.15 (6.62–7.50)	7.19 (6.36–7.66)	0.164
Hematocrit (%)	42.80 (38.80–47.00)	44.75 (39.60–49.60)	45.00 (43.20–47.60)	44.90 (39.70–48.20)	0.223
MCV (fL)	63.80 (61.80–64.95)	64.90 (62.33–66.70)	63.90 (60.30–67.40)	63.60 (60.90–65.50)	0.327
MCH (pg)	24.29 (23.55–24.90)	23.75 (22.00–24.63)	21.00 (20.50–22.90)^a,b^	22.40 (20.90–23.20)^a,b^	<0.001
MCHC (g/dL)	38.20 (37.50–39.05)	36.55 (33.53–37.78) ^a^	32.90 (32.00–35.00)^a^	35.30 (33.30–36.80)^a^	<0.001
RDW (%)	14.00 (13.05–14.55)	13.70 (13.08–14.25)	13.30 (12.80–14.50)	14.10 (13.20–14.90)	0.383
Platelet count (×10^3^ cells/μL)	283.00 (228.50–341.00)	315.50 (229.00–386.75)	346.00 (290.00–413.00)	328.00 (249.00–432.00)	0.167
MPV (fL)	10.10 (9.05–11.35)	11.05 (10.25–11.88)	10.90 (10.20–11.90)	10.50 (9.50–11.30)	0.098
PDW (%)	15.30 (15.10–15.50)	15.35 (15.23–15.68)	15.10 (14.90–15.40)	15.10 (15.00–15.30) ^a,b^	0.001
WBC (×10^3^ cells/μL)	8.37 (6.98–10.82)	8.33 (7.21–10.80)	8.41 (7.40–9.37)	8.74 (7.52–11.30)	0.651
Neutrophil (×10^3^ cells/μL)	5.87 (4.05–7.31)	6.14 (4.14–8.02)	6.08 (5.06–7.71)	6.21 (4.97–7.66)	0.595
Eosinophil (×10^3^ cells/μL)	0.31 (0.20–0.30)	0.29 (0.22–0.48)	0.26 (0.21–0.34)	0.34 (0.22–0.56)	0.439
Basophil (×10^3^ cells/μL)	0.02 (0.01–0.03)	0.01 (0.00–0.03)	0.00 (0.00–0.01) ^a^	0.01 (0.00–0.02) ^a^	0.005
Lymphocyte (×10^3^ cells/μL)	1.53 (1.18–1.94)	1.43 (1.06–1.86)	1.47 (1.21–2.28)	1.28 (1.03–1.70)	0.196
Monocyte (×10^3^ cells/μL)	0.57 (0.44–0.83)	0.62 (0.35–0.90)	0.32 (0.17–0.56) ^a^	0.53 (0.29–0.75)	0.015
LA (cm/kg)	0.92 (0.81–1.04)	1.30 (0.98–1.66)	1.71 (1.62–2.25) ^a^	2.17 (1.85–2.59) ^a,b^	<0.001
Ao (cm/kg)	0.76 (0.68–0.86)	0.88 (0.74–1.38)	1.18 (0.89–1.31) ^a^	1.16 (0.97–1.31) ^a^	<0.001
LA/Ao	1.20 (1.11–1.35)	1.37 (1.13–1.49)	1.70 (1.46–2.03) ^a^	1.88 (1.59–2.37) ^a,b^	<0.001
IVSd (cm/kg)	0.47 (0.40–0.52)	0.41 (0.38–0.50)	0.44 (0.42–0.48)	0.43 (0.38–0.49)	0.152
LVIDd (cm/kg)	1.22 (1.17–1.37)	1.30 (1.25–1.45)	1.80 (1.55–1.94) ^a,b^	1.78 (1.60–1.99) ^a,b^	<0.001
LVPWd (cm/kg)	0.38 (0.33–0.43)	0.40 (0.36–0.45)	0.45 (0.35–0.50)	0.39 (0.36–0.45)	0.235
IVSs (cm/kg)	0.61 (0.52–0.67)	0.60 (0.51–0.67)	0.70 (0.59–0.76)	0.64 (0.55–0.73)	0.027*
LVIDs (cm/kg)	0.74 (0.62–0.80)	0.79 (0.62–0.90)	0.91 (0.67–0.98)	0.90 (0.76–1.06) ^a^	<0.001
LVPWs (cm/kg)	0.64 (0.56–0.71)	0.62 (0.54–0.69)	0.66 (0.61–0.76)	0.69 (0.61–0.76)	0.025*
%FS	39.86 (34.75–45.68)	39.80 (31.97–48.43)	49.17 (45.47–54.48)^a^	48.85 (43.48–53.51)^a,b^	<0.001

Ao, aorta; FS, fractional shortening; IVSd, interventricular septal thickness at end diastole; IVSs, interventricular septal thickness at end systole; LA, left atrium; LA/Ao, the ratio of the left atrial dimension to the aortic annulus dimension; LVIDd, left ventricular internal diameter at end diastole; LVIDs, left ventricular internal diameter at end systole; LVPWd, left ventricular posterior wall thickness at end diastole; LVPWs, left ventricular posterior wall thickness at end systole; MCH, mean corpuscular hemoglobin; MCHC, mean corpuscular hemoglobin concentration; MCV, mean corpuscular volume; MPV, mean platelet volume; PDW, platelet distribution width; RBC, red blood cell; RDW, red cell distribution width; WBC, white blood cell.

The *p*-values represent the significant difference among four groups by the Kruskal-Wallis test.

*No significant difference was found after analysis with Dunn’s test.

^a^Indicates significant difference from the normal group.

^b^Indicates significant difference from the MMVD B1 group.

### Analysis of red blood cell and platelet indices

**Figure 1:**
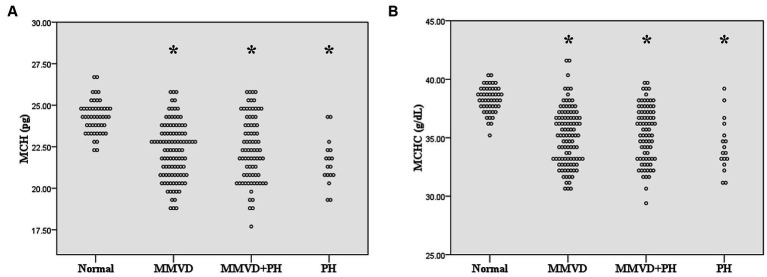
Dot plot of **(A)** mean corpuscular hemoglobin (MCH) and **(B)** mean corpuscular hemoglobin concentration (MCHC) of normal dogs (*n* = 49), dogs with MMVD (*n* = 102), MMVD dogs with PH (*n* = 78), and PH dogs without MMVD (*n* = 17). The MCH and MCHC of all disease groups were less than normal dogs (*p* < 0.001). The single asterisk presents a significant difference from the normal group.

**Figure 2:**
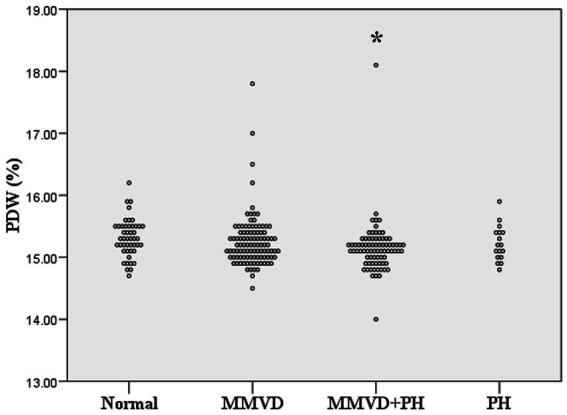
Dot plot of platelet distribution width (PDW) of normal dogs (*n* = 49), dogs with MMVD (*n* = 102), MMVD dogs with PH (*n* = 78), and PH dogs without MMVD (*n* = 17). The PDW of normal dogs was greater than those of MMVD dogs with PH (*p* = 0.04). The single asterisk presents a significant difference from the normal group.

**Figure 3:**
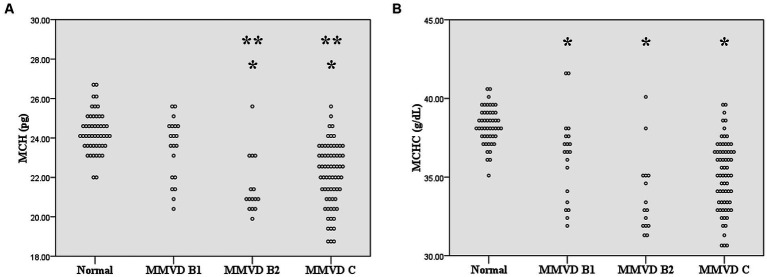
Dot plot of mean corpuscular hemoglobin (MCH) and mean corpuscular hemoglobin concentration (MCHC) of normal dogs (*n* = 49), dogs with stage B1 MMVD (*n* = 20), dogs with stage B2 MMVD (*n* = 15), and dogs with stage C MMVD (*n* = 67). **(A)** MCH of dogs with stage B2 and C MMVD groups were lower than those of normal dogs (*p* < 0.001) and dogs with stage B1 MMVD (*p* = 0.006 and *p* < 0.001 respectively). **(B)** MCHC of dogs with stage B1 (*p* = 0.004), B2 (*p* < 0.001) and C MMVD (*p* < 0.001) were lower than that of normal dogs. The single asterisk presents a significant difference from the normal group. The double asterisk presents a significant difference from the MMVD B1 group.

**Figure 4:**
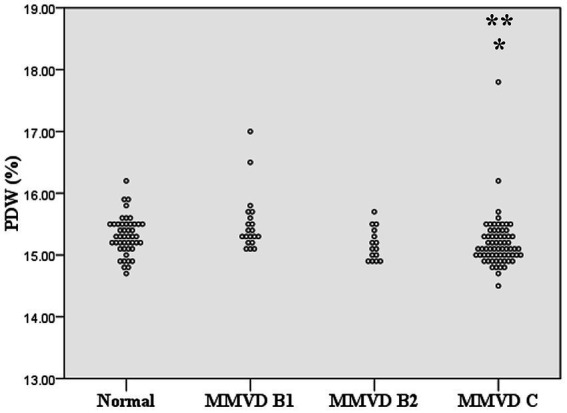
Dot plot of platelet distribution width (PDW) of normal dogs (*n* = 49), dogs with stage B1 MMVD (*n* = 20), dogs with stage B2 MMVD (*n* = 15), and dogs with stage C MMVD (*n* = 67). The PDW of dogs with stage C MMVD was lower than those of normal dogs (*p* = 0.042) and dogs with stage B1 MMVD (*p* = 0.02). The single and double asterisk presents a significant difference from the normal group and stage B1 MMVD, respectively.

No difference in MCV and RDW was observed among groups. MCH and MCHC of dogs in MMVD, MMVD+PH, and PH groups were lower than those of normal dogs (*p* < 0.001) (Table 1 and Figure 1). Comparison of neither MCH nor MCHC between the disease groups revealed any significant difference. Analysis of platelet indices, MPV and PDW, revealed lower PDW in MMVD+PH groups compared to the normal group (*p* = 0.004) (Table 1 and Figure 2). There was no significant difference in PDW, neither in comparison between MMVD+PH and MMVD groups nor in comparison between MMVD+PH and PH groups. The results of hematologic profiles are shown in Table 1. To clarify whether the changes in MCH, MCHC, and PDW were associated with MMVD, a comparison of MCH, MCHC, and PDW between the different stages of MMVD was performed in dogs that were included in the MMVD group from the first analysis. The dogs in stage B2 had lower MCH compared to normal dogs (*p* < 0.001) and dogs in stage B1 MMVD (*p* = 0.006). The dogs in stage C MMVD had lower MCH compared to normal dogs (*p* < 0.001) and dogs in stage B1 MMVD (*p* = 0.009). MCHC of normal dogs was greater than dogs with stage B1 (*p* = 0.004), B2 (*p* < 0.001), and C (*p* < 0.001) (Table 3 and Figure 3). The dogs with stage C MMVD had lower PDW compared to normal dogs (*p* = 0.042) and dogs with stage B1 MMVD (*p* = 0.002) (Table 3 and Figure 4). Analysis of WBC revealed no significant difference among the groups, except for lower monocyte count in dogs with stage B2 MMVD compared to the normal group (*p* = 0.010) (Table 3). The results of hematological profiles of all dogs are shown in Tables 1, 3.

**Figure 5:**
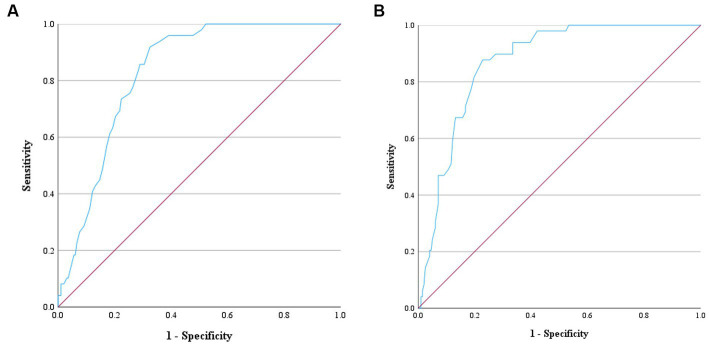
Receiver operating characteristic (ROC) curve for ascertaining the prediction of MCH and MCHC to distinguish normal dogs from dogs with MMVD and/or PH. The positive actual state is normal dogs. **(A)** ROC curve of MCH showed the area under the curve (AUC) value of 0.83 and the cut-point value of 23.05 pg. Therefore, dogs with MCH > 23.05 pg. can be predicted as normal dogs with the sensitivity of 91.80% and specificity of 67.50%. **(B)** ROC curve of MCHC showed the AUC value of 0.87 and the cut-point value of 37.05 g/dL. Therefore, dogs with MCHC>37.05 g/dL can be predicted as normal dogs with the sensitivity of 87.80% and specificity of 77.20%.

The sensitivity and specificity for discriminating between normal dogs and diseased dogs including dogs with MMVD (both with and without PH), as well as dogs with PH from other causes, were 91.80 and 67.50%, respectively, for MCH, and 87.80 and 77.20%, respectively for MCHC. The AUC values of MCH and MCHC were 0.83 and 0.87, indicating a discriminative value in distinguishing between normal dogs and those with MMVD. The optimal cut-off values 23.05 pg. for MCH, and 37.05 g/dL for MCHC (Figure 5). However, PDW values were unable to differentiate between normal dogs and diseased dogs.

Greater blood urea nitrogen (BUN) was found in dogs with MMVD (*p* < 0.001) and MMVD+PH (*p* < 0.001) compared to the normal dogs. Moreover, sub-analysis in MMVD dogs showed that the stage C MMVD dogs had greater BUN compared to normal dogs (*p* < 0.001) and stage B1 MMVD dogs (*p* = 0.011). Creatinine levels of MMVD+PH group were higher than those in PH group (*p* = 0.010). Sub-analysis of MMVD dogs showed that creatinine levels in stage C MMVD dogs were also higher than those in stage B1 MMVD dogs (*p* = 0.010). Comparison of the albumin level to normal dogs revealed a decrease in albumin levels in all disease groups (*p* < 0.001). The blood chemical profiles of all dogs are presented in Tables 2, 4.

**Table 4:** Blood chemistry profiles of dogs with myxomatous mitral valve disease.

	Normal (*n* = 49)	MMVD B1 (*n* = 20)	MMVD B2 (*n* = 15)	MMVD C (*n* = 67)	*P-*value
ALT (U/L)	45.00 (34.00–56.50)	49.00 (30.00–66.00)	50.00 (30.00–79.00)	58.00 (38.00–86.00)^a^	0.013
ALP (U/L)	50.00 (24.00–87.00)	55.00 (27.00–84.00)	51.00 (29.00–100.00)	58.00 (39.50–107.00)	0.556
BUN (mg/dL)	17.60 (13.95–23.25)	19.00 (13.40–27.60)	24.00 (19.10–29.30)	27.35 (22.25–37.60)^a,b^	<0.001
Creatinine (mg/dL)	0.80 (0.70–0.90)	0.70 (0.50–0.80)	0.70 (0.60–0.90)	0.85 (0.68–1.10)^b^	0.014
Total protein (g/dL)	6.40 (6.00–6.90)	6.60 (6.30–6.80)	6.40 (6.10–6.80)	6.70 (6.50–7.20)^a^	0.005
Albumin (g/dL)	3.20 (3.00–3.50)	3.00 (2.70–3.50)	2.50 (2.40–2.70)^a,b^	2.80 (2.60–3.20)^a,c^	<0.001

Values are median (interquartile).

ALP, alkaline phosphatase; ALT, alanine aminotransferase; BUN, blood urea nitrogen.

^a^Indicates significant difference from the normal group.

^b^Indicates significant difference from the MMVD B1 group.

^c^Indicates significant difference from the MMVD B2 group.

**Figure 6:**
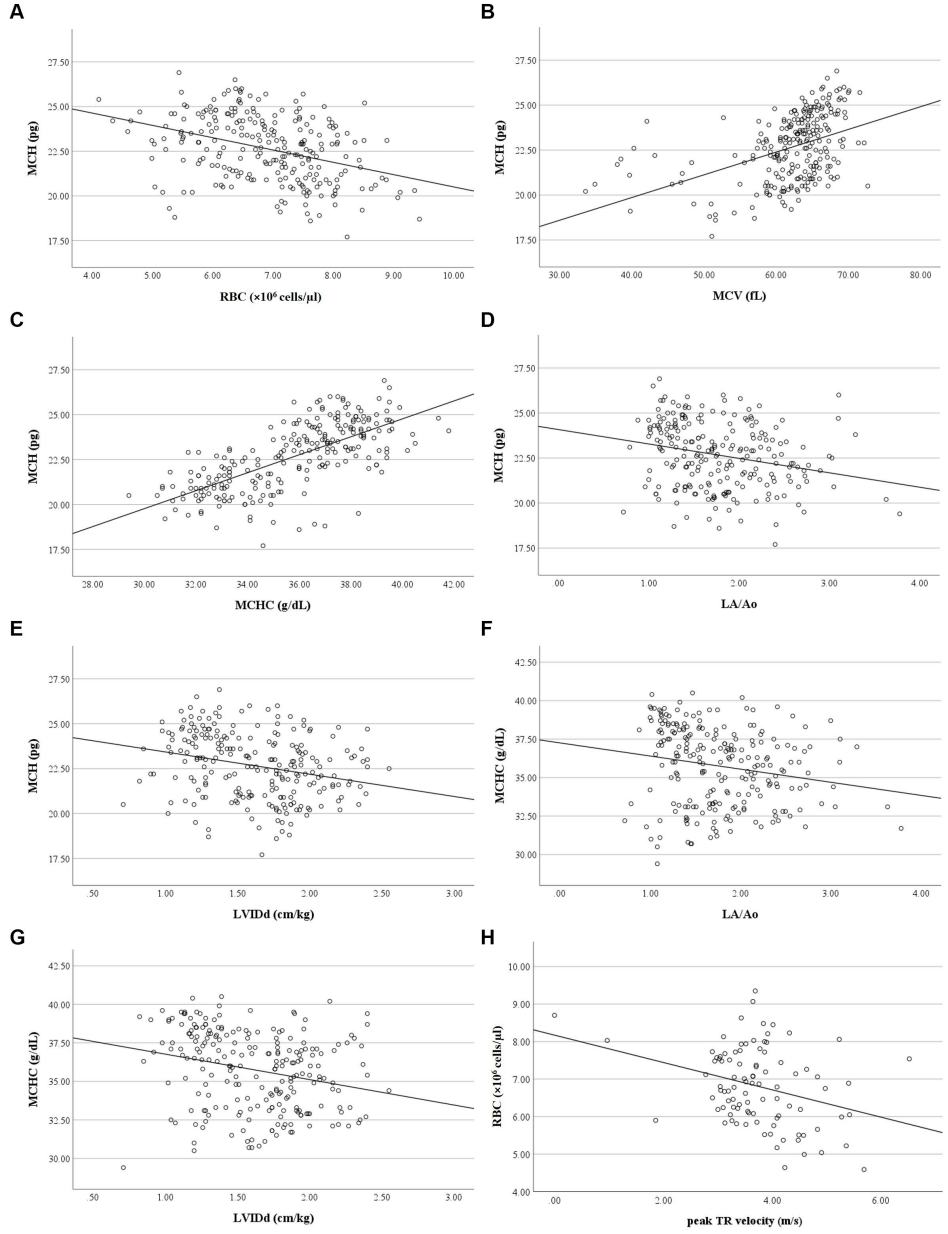
Correlation of RBC indices, RBC count, LA/Ao LVIDd and peak TR velocity. **(A)** MCH was negatively correlated with RBC count (*r* = −0.377, *p* < 0.001). **(B)** MCH was positively correlated with MCV (*r* = 0.484, *p* < 0.001). **(C)** MCH was positively correlated with MCHC (*r* = 0.717, *p* < 0.001). **(D)** MCH was negatively correlated with LA/Ao (*r* = −0.263, *p* < 0.001). **(E)** MCH was negatively correlated with LVIDd (*r* = −0.291, *p* < 0.001). **(F)** MCHC was negatively correlated with LA/Ao (*r* = −0.237, *p* < 0.001). **(G)** MCHC was negatively correlated with LVIDd (*r* = −0.266, *p* < 0.001). **(H)** RBC count was negatively correlated with peak TR velocity (*r* = −0.279, *p* = 0.006).

**Figure 7:**
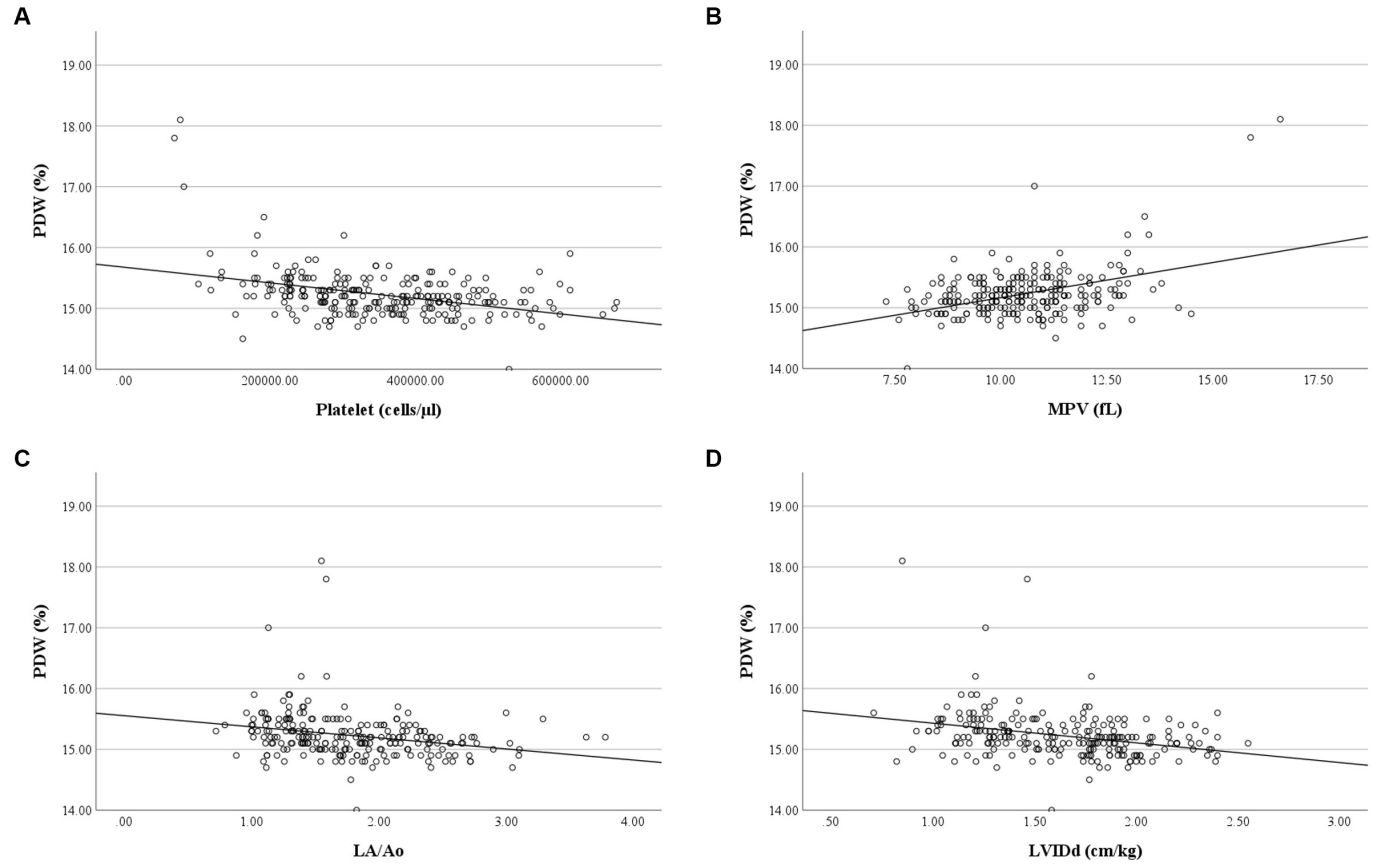
Correlation of PDW and platelet count, MPV, LA/Ao and LVIDd. **(A)** PDW was negatively correlated with platelet count (*r* = −0.317, *p* < 0.001). **(B)** PDW was positively correlated with MPV (*r* = 0.233, *p* < 0.001). **(C)** PDW was negatively correlated with LA/Ao (*r* = −0.304, *p* < 0.001). **(D)** PDW was negatively correlated with LVIDd (*r* = −0.312, *p* < 0.001).

Correlation analysis revealed no relationship between RBC and platelet indices, and age, weight, and breed of the dogs in the present study. MCH had a weak negative correlation with RBC count (*r* = −0.38, *p* < 0.001), a moderate positive correlation with MCV (*r* = 0.48, *p* < 0.001), and a strong positive correlation with MCHC (*r* = 0.72, *p* < 0.001). Weak negative correlations were found between MCH and LA/Ao (*r* = −0.26, *p* < 0.001) and LVIDd (*r* = −0.29, *p* < 0.001), as well as weak correlations between MCHC and LA/Ao (*r* = −0.24, *p* < 0.001) and LVIDd (*r* = −0.27, *p* < 0.001). RBC count had a weak negative correlation with peak TR velocity and calculated PAP (*r* = −0.279, *p* = 0.006) (Figure 6). PDW of dogs in the present study had a weak positive correlation with MPV (*r* = 0.23, *p* < 0.001) but had a weak negative correlation with platelet count (*r* = −0.32, *p* < 0.001), LA/Ao (*r* = −0.30, *p* < 0.001), and LVIDd (*r* = −0.31, *p* < 0.001) (Figure 7).

## Discussion

Pulmonary hypertension due to respiratory problems and heartworm disease is classified as precapillary PH, while PH secondary to MMVD is postcapillary PH (1). A previous study found increased RDW in dogs with precapillary PH compared to normal dogs but not in dogs with post-capillary PH (30). On the other hand, another study revealed increased RDW in both dogs with precapillary and postcapillary PH compared to normal dogs (31). However, an increase in RDW was not associated with the severity of PH (31, 35). In this study, we found no difference in RDW among normal and dogs with precapillary and postcapillary PH. The reason for RDW alteration remains unclear in both human patients and dogs with PH. Besides, RDW can be altered by other diseases and conditions including immune-mediated diseases, hormonal diseases, lung worms, pulmonary fibrosis, thromboembolism, systemic-to-pulmonary shunts, aging, and unknown causes (30, 31, 36). Therefore, concurrent diseases may also affect RDW. However, we made efforts to exclude dogs with other diseases from the study. Although an increase in RDW values was found in dogs with several diseases and conditions, the values were still within the normal reference interval.

MCV reflects the RBC size, while MCH and MCHC define the weight and concentration of hemoglobin per RBC, respectively, (23). Decreases in MCH and MCHC were found in human patients with pulmonary arterial hypertension compared to the healthy controls, and these changes may be affected by decreases in RBC count, hemoglobin and hematocrit, and an increase in RDW (24). Moreover, decreased MCHC or hypochromasia in anemic dogs may indicate iron deficiency (22). In the present study, MCH and MCHC were reduced in MMVD, MMVD+PH, and PH groups compared to the normal dogs. Even though the AUC values of the ROC curves of these two indices suggest moderate discriminatory power, caution might be warranted when considering their use as markers for distinguishing between normal dogs, dogs with MMVD (with and without PH), and dogs with PH from other causes. This is due to the fact that the values of MCH and MCHC remained within the normal limits for all groups of dogs, whether they were normal or diseased. Furthermore, MCH was found to be correlated to RBC count and MCV. However, no differences in RBC count, MCV and RDW were observed among the groups. This evidence indicates that MMVD and PH may alter the concentration of hemoglobin in dogs, but these conditions may not affect the size and number of RBCs. A study in humans demonstrated that anemia was commonly observed in patients with advanced stages of PH (37). In current study, weak negative correlations were observed between RBC count and both peak TR velocity and estimated PAP. Furthermore, the RBC count for all groups in the present study fell within the normal reference range, and there were no differences in RBC count among the groups. Collectively, it can be inferred that the severity of PH, as assessed by TR velocity and estimated PAP, may have a weak relationship with RBC numbers, which may not hold clinical significance.

Increased MPV has been detected in human patients with mitral stenosis (38), mitral regurgitation (13), ischemic heart disease (29), and PH (8, 11, 14). On the other hand, decreased MPV was found in children with congenital heart disease and PH compared to those without PH (39). In this study, no change in MPV was detected in dogs with MMVD without PH, MMVD with PH (post-capillary PH) and PH due to other causes (pre-capillary PH). Due to the conflicting findings in humans and dogs, the usefulness of MPV as an indicator for monitoring MMVD and PH in dogs is uncertain.

In the present study, a reduction in PDW was observed in the MMVD+PH groups compared with the normal group. However, there was no difference in PDW between MMVD dogs with and without PH. To clarify whether decreased PDW was related to MMVD, we assessed PDW according to the severity of MMVD. A decrease in PDW was found in dogs with stage C MMVD, along with a negative correlation between PDW and left atrial and ventricular size without correlation with peak TR velocity. Therefore, decreased PDW may be related to the severity of MMVD, but not the occurrence of PH because the change in PDW was not found in dogs with PH secondary to MMVD and other causes. Previous studies showed that PDW was increased in human patients with PH, and positively correlated with left ventricular hypertrophy and dysfunction (14, 28). In another previous study, children with PH due to congenital heart disease were found to have lower PDW than children without PH (39). In general, PDW reflects variation in platelet sizes and platelet activation (40). Therefore, a decrease in PDW may indicate that activated platelets are being consumed or destroyed in blood vessels (39). Based on the result of the present study, it is speculated that the platelet consumption and/or destruction was increased in dogs with MMVD with increased severity. Platelet fragmentation and decreased function can be caused by shear stress from mitral regurgitation (41, 42). However, this event may not relate to PH in dogs. Further investigation of the role of platelets in canine MMVD and PH is needed to improve the understanding of the association between platelets and MMVD and PH.

This study investigated the changes in RBC and platelet indices in dogs with PH. Because many diseases, including neoplasia and inflammatory diseases in other organs, can affect RBC and platelet indices, data of dogs with these concurrent diseases were excluded in this study (27, 43, 44). A significant age difference was found between the normal group and the other groups. However, there was no correlation between age and RBC and platelet indices suggesting that age may not affect these indices.

Monocytes are the mononuclear cells that play an essential role in the immune response. In addition to their involvement in immunity, monocytes also have a part in inflammation and tissue remodeling, including myocardial tissue (45). A previous study reported that monocytes were increased in dogs with CHF compared to control dogs, which may indicate cardiac remodeling in heart failure (46). Monocyte-to-lymphocyte ratio (MLR) is one of the inflammatory markers indicating the severity of heart diseases such as, human myocarditis (47). A greater MLR was revealed in dogs with CHF due to MMVD (48). However, this study found a decreased monocyte count of dogs with stage B2 MMVD compared to the normal dogs, while no difference of monocyte count between stage C MMVD and normal dogs was found. This finding is not consistent with previous studies. Therefore, the change in monocyte count of dogs with stage B2 MMVD in the present study may have occurred accidentally and does not provide clinical significance.

In the present study, alterations of BUN and albumin levels were found. BUN levels were elevated in dogs with MMVD and MMVD+PH compared to the normal dogs, and sub-analysis in MMVD dogs revealed that BUN levels were increased in stage C MMVD dogs. This finding is in accordance with a previous study in dogs with precapillary and postcapillary PH, where BUN concentration was increased without an elevation of creatinine levels (31). Increased BUN in heart disease can be a complication of CHF (49). Furthermore, the treatment of CHF using the diuretics and angiotensin converting enzyme inhibitors can cause an increase in BUN levels (31, 50). Creatinine levels of dogs enrolled in the present study were increased in MMVD+PH dogs compared to PH dogs, and stage C MMVD dogs compared to stage B1 MMVD dogs. These findings indicated that elevated creatinine levels were found in dogs with CHF due to MMVD. Azotemia and decreased glomerular filtration rate can be found in dogs with advanced stage of chronic valvular disease and associated with the severity of the disease (51). For this reason, greater BUN and creatinine levels can arise from CHF owing to progressive MMVD.

Decreased albumin levels were found in dogs with MMVD, MMVD+PH and precapillary PH, and sub-analysis of MMVD dogs showed that low albumin levels were related to severity of the disease. Several diseases and abnormalities including chronic heart failure and PH, were associated with low levels of albumin as a result of hemodilution caused by volume overload (52). Consequently, the evidence of decreased BUN and albumin in dogs with CHF and PH may reveal the progression of MMVD and PH.

A limitation of the study is the small sample size, which may affect the statistical significance of the RBC and platelet indices. A larger number of dogs could provide more accurate results, depicting the changes in these indices in dog populations.

In conclusion, the present study found that the decrease in PDW of dogs with MMVD was related to the severity of MMVD but not PH. No changes in platelet indices were not found in dogs with PH from other causes when compared to normal dogs. However, the RBC indices, MCH and MCHC, were associated with MMVD, precapillary and postcapillary PH. Further investigation of the role of RBC and platelets in canine MMVD and PH may improve the understanding of the association between PDW and MMVD and PH.

## Data availability statement

The raw data supporting the conclusions of this article will be made available by the authors, without undue reservation.

## Ethics statement

The requirement of ethical approval was waived by the Chulalongkorn University Animal Care and Use Committee for the studies involving animals because the design of the study was retrospective. The studies were conducted in accordance with the local legislation and institutional requirements.

## Author contributions

NT was responsible for data collection, data analysis, and manuscript writing and revision. EO was responsible for manuscript revision and editing. SS was responsible for data analysis and manuscript revision and editing. All authors contributed to the article and approved the submitted version.

## Funding

This research project was supported by the Secondary Century Fund (C2F), Chulalongkorn University and the research grant form Faculty of Veterinary Science, Chulalongkorn University.

## Conflict of interest

The authors declare that the research was conducted in the absence of any commercial or financial relationships that could be construed as a potential conflict of interest.

## Publisher’s note

All claims expressed in this article are solely those of the authors and do not necessarily represent those of their affiliated organizations, or those of the publisher, the editors and the reviewers. Any product that may be evaluated in this article, or claim that may be made by its manufacturer, is not guaranteed or endorsed by the publisher.

## Figures and Tables

**Table:** 

Ao	Aorta
ALP	Alkaline phosphatase
ALT	Alanine aminotransferase
BUN	Blood urea nitrogen
CHF	Congestive heart failure
FS	Fractional shortening
IQR	Interquartile range
IVSd	Interventricular septal thickness at end diastole
IVSs	Interventricular septal thickness at end systole
LA	Left atrium
LVIDd	Left ventricular internal diameter at end diastole
LVIDs	Left ventricular internal diameter at end systole
LVPWd	Left ventricular posterior wall thickness at end diastole
LVPWs	Left ventricular posterior wall thickness at end systole
MCH	Mean corpuscular hemoglobin
MCHC	Mean corpuscular hemoglobin concentration
MCV	Mean corpuscular volume
MMVD	Myxomatous mitral valve disease
MPV	Mean platelet volume
PAP	Pulmonary arterial pressure
PDW	Platelet distribution width
PH	Pulmonary hypertension
RBC	Red blood cell
RDW	Red cell distribution width
TR	Tricuspid regurgitation
WBC	White blood cell
